# Inhibitory effect of dimethyl sulfoxide on the development of gastrointestinal nematode larvae in the larval development test

**DOI:** 10.2478/jvetres-2025-0016

**Published:** 2025-03-25

**Authors:** Marcin Mickiewicz, Zofia Nowek, Michał Czopowicz, Agata Moroz-Fik, Adrian-Valentin Potărniche, Kinga Biernacka, Olga Szaluś-Jordanow, Paweł Górski, Alistair Antonopoulos, Iwona Markowska-Daniel, Marián Várady, Jarosław Kaba

**Affiliations:** Division of Veterinary Epidemiology and Economics; Department of Infectious Diseases and Preventive Medicine, Law and Ethics, University of Agricultural Sciences and Veterinary Medicine, Cluj-Napoca 400372, Romania; Department of Small Animal Diseases with Clinic, Institute of Veterinary Medicine, Warsaw University of Life Sciences–SGGW, 02-776 Warsaw, Poland; Division of Parasitology and Invasiology, Department of Preclinical Sciences, Institute of Veterinary Medicine, Warsaw University of Life Sciences–SGGW, 02-786 Warsaw, Poland; Kreavet, 9150 Kruibeke, Belgium; Institute of Parasitology, Slovak Academy of Sciences, 04001 Košice, Slovakia

**Keywords:** anthelmintic resistance, DMSO, five-parameter logistic curve, inhibitory concentration, Trichostrongylidae

## Abstract

**Introduction:**

Dimethyl sulfoxide (DMSO) is an amphipathic solvent for molecules in *in vitro* tests for detection of anthelmintic resistance of gastrointestinal nematodes (GIN). It has been shown to have a concentration-dependent detrimental effect on *Caenorhabditis elegans*, a free-living nematode. If GIN are likewise affected, using DMSO in egg-hatch test and larval development test (LDT) may confound their results. Therefore, the DMSO concentration was determined at which it exerted an inhibitory effect on GIN larval development to the third stage.

**Material and Methods:**

A standard LDT was performed in 30 replications at DMSO concentrations of 0.0% (control), 0.6%, 1.3%, 2.6%, 5.2%, 10.4%, and 20.8%. The numbers of all developmental stages of *Haemonchus contortus, Trichostrongylus* spp. and *Oesophagostomum* spp. (unhatched eggs, larvae of the first, second and third stages (L1-L3) were determined, the proportion of L3 (the percentage of larval development – PD) was calculated and L3 were identified at the species or genus level. A five-parameter logistic curve was fitted to the observed PDs and modelled the DMSO–larval development relationship.

**Results:**

The PD significantly decreased with increasing DMSO concentration and was significantly reduced at the 2.6% concentration. The median inhibitory concentration (IC50) was 3.79%, the concentration for 10% inhibition (IC10) was 1.75% and for 90% inhibition (IC90) was 8.20%. The percentage of L1 and L2 followed an analogical but opposite pattern to that of PD and was complementary to it at each DMSO concentration. The unhatched egg percentage was rarely >1% and showed no pattern.

**Conclusion:**

At ≥2.6% concentration, DMSO significantly inhibited the L3 development of all three GIN species. It had a practically important inhibitory effect (IC10) at as low concentration as 1.75%. At lower concentrations, DMSO did not appear to inhibit larval development. The compound did not seem to exert an *in vitro* ovicidal effect regardless of the concentration.

## Introduction

Dimethyl sulfoxide (DMSO) is produced in nature and is known to be a source of carbon and sulphur for marine microorganisms (27). This polar aprotic solvent does not contain hydrogen or nitrogen in its structure and is soluble in both aqueous and organic environments (4, 32). The amphipathic nature of DMSO makes it highly useful for dissolving polar and nonpolar molecules (45). It is often used in pharmacology and toxicology to enhance the solubility of poorly soluble polar and nonpolar molecules, *e.g*. for developmental toxicity testing of chemicals and pharmaceuticals in zebrafish embryos, and in *in vitro* tests for detection of anthelmintic resistance (AR) of gastrointestinal nematodes (GIN) such as the larval development test (LDT) and egg hatch test (EHT) (8, 17, 21, 40). Most commonly used anthelmintics such as benzimidazoles (BZ) and macrocyclic lactones (ML) are poorly soluble in water and other solutions of known neutral effect on the GIN larvae (6, 36), but they are highly soluble in DMSO (26, 50). Over the previous decade, a number of studies have shown that this solvent has a concentration-dependent detrimental effect on living organisms which can affect the results of the laboratory procedures for which it is used (5, 12, 27, 39). These negative effects include reduced fertility, lifespan or feeding and are well documented in the case of the nematode *Caenorhabditis elegans*, which serves as a model organism in pharmacological experiments (1, 11, 16, 19). Some studies have also described morphological changes in the internal body composition of *C. elegans* following prolonged exposure to DMSO, which arise over time in drug screening LDT and EHT (5). Therefore, when DMSO is used in *in vitro* drug exposure tests, these confounding effects of the vehicle should be considered when analysing and interpreting results. The evidence of these effects has been gathered mostly from the *C. elegans* model, the physiology and life cycle of which differ from those of parasitic nematodes (47). Studies investigating the effects of DMSO on parasitic nematodes are still lacking (49).

In recent decades several *in vivo* and *in vitro* tests have been developed for AR detection in GIN. Among the *in vitro* techniques, the EHT and LDT are the most widely employed (9, 44) and diagnose AR in pre-parasitic stages. Both tests are able to detect resistance to BZ, and the LDT also detects resistance to ML and levamisole (LEV) (9, 13, 14, 43). Even though the LDT can be performed in two versions involving the use of liquid (23) or agar culture media (30), the basic principle remains the same. The eggs are cultured to the infective third larval stage (L3) at increasing anthelmintic concentrations. The LDT (liquid or agar-based) is the most broadly applied *in vitro* test for AR screening studies (2, 3, 13, 15, 20, 25, 29, 34, 35, 37, 38). In both LDT versions, BZ and ML are dissolved in DMSO, and only LEV is dissolved in distilled water (8, 9, 23, 30). The common use of DMSO as a solvent (vehicle) of anthelmintics in both versions of the LDT does not extend to a common final concentration of DMSO to be used. The recommended concentrations differ between versions and studies but the final concentration never exceeds 2% (9, 17, 28, 35, 38, 44). However, no data available in the literature indicate the highest DMSO concentration which can be used in the LDT without any negative effect on the test results. Therefore, a laboratory experiment was carried out to determine the highest safe DMSO concentration at which it can be used as the solvent of anthelmintics in the LDT.

## Material and Methods

A liquid-based LDT was performed according to the technique described by Hubert and Kerboeuf (23) with the modification of Várady *et al*. (42, 43). A fresh faecal sample was collected directly from the rectum of two naturally infected adult goats with a mean faecal egg count of 1,050 per gram (epg). Eggs were collected by passing the fresh faecal sample through three stacked sieves with apertures of 250, 100 and 25 μm. The material collected on the 25 μm sieve was washed with tap water and sedimented, and then the trichostrongylid eggs were recovered by the salt flotation method with saturated sodium chloride (8). After extraction, eggs were inspected microscopically to ensure that embryonation had not yet begun and suspended in deionised water at a concentration of 70–100 eggs per 10 μL per well. Stock DMSO solutions (Sigma-Aldrich, Darmstadt, Germany) were prepared by serial dilutions of a ≥99.9% pure reagent in deionised water to yield six final concentrations of 0.6%, 1.3%, 2.6%, 5.2%, 10.4% and 20.8% (v/v) in the tested wells; a 0.0% concentration (pure deionised water) was used in the control wells. The LDT was performed on 96-well cell culture plates (Sarstedt, Nűmbrecht, Germany) in 150 μL of culture medium which consisted of 10 μL of a particular DMSO stock solution or a deionised water (control wells), 110 μL of deionised water, 20 μL of culture medium as described by Hubert and Kerboeuf (22) and 10 μL of a suspension (approximately 70-100 eggs) containing amphotericin B (Sigma-Aldrich, Darmstadt, Germany) at a concentration of 5 μg/mL. Each DMSO concentration was examined in 30 replications. The plates were sealed to prevent drying and incubated at 25°C for 7 days. After the incubation period, 10 μL of Lugol’s solution was added to each well to terminate the test. The number of unhatched eggs and larvae of the first and second stages (L1 and L2) in each well were counted under an inverted microscope (Eclipse Ts2; Nikon, Tokyo, Japan). The L3 were identified at the species or genus level according to the procedure described by van Wyk and Mayhew (41). The percentage of larval development (PD), defined as the proportion of L3 in all developmental stages observed in the well, was calculated at each DMSO concentration and in control wells. All the procedures were performed by the same examiner (MM).

To confirm the species/generic composition of GIN present in faeces, a larval culture was prepared by mixing 5 g of faeces collected from the two goats into one pooled sample. After Baermannisation and addition of 10 μL of Lugol’s solution, 100 L3 were identified at the genus or species level in a light microscope (Nikon Eclipse E200) at 500× magnification according to the procedure described by Wyk and Mayhew (41), and the percentage of each nematode genus/species was determined. *Trichostrongylus* spp. and *Teladorsagia circumcincta* were differentiated after exsheathment of the L3 in 3.5% sodium hypochlorite solution (Chempur, Piekary Śląskie, Poland) by comparing specific morphological features of these two GIN genera (33). The susceptibility to anthelmintics of the field GIN strains used in the study was unknown.

The percentages of particular developmental stages (eggs, L1 and L2, and L3) at increasing DMSO concentrations were presented as the arithmetic mean, standard deviation (±SD), and range or the arithmetic mean and 95% confidence interval on the graph. They were compared with the PD in control wells using one-way analysis of variance followed, if significant, by Dunnett’s post-hoc test. The concentrations inhibiting development of 10% (inhibitory concentration; IC10), 50% (IC50) and 90% (IC90) of L3 were estimated by fitting a five-parameter logistic curve (5PL) to the mean PDs at subsequent DMSO concentrations. The fitting of the 5PL curve was based on minimisation of the sum of squared errors weighted by the inverse variance of the 30 responses at six subsequent DMSO concentrations (18). The PD at 0% DMSO concentration (control wells) was used to set the upper asymptote (d) of the 5PL curve. The proportions of L3 of *Haemonchus contortus* and *Trichostrongylus* spp. were compared using McNemar’s test. All statistical tests were two-tailed. A significance level (α) was set at 0.05 and the Bonferroni correction was used in the case of multiple comparisons of proportions. The analysis was performed in TIBCO Statistica 13.3 (TIBCO Software Inc., Palo Alto, CA, USA).

## Results

The number of eggs distributed to wells ranged from 60 to 104 (71 ± 9) and did not differ significantly either between DMSO concentrations (P = 0.300) or between replications (P = 0.952).

**Fig. 1.**
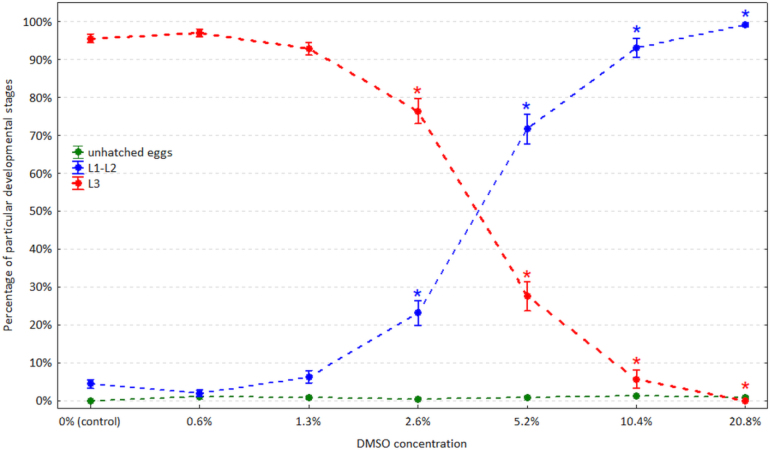
The observed percentage of unhatched eggs, larvae of the first and second stage (L1-L2) and larvae of the third stage (L3; percentage development) of *Haemonchus contortus, Trichostrongylus* spp. and *Oesophagostomum* spp. at increasing dimethyl sulfoxide (DMSO) concentrations. Dots – arithmetic means; whiskers – 95% confidence intervals; asterisks (*) – statistically significantly lower percentages than in the control wells according to Dunnett’s test at α = 0.05

**Fig. 2.**
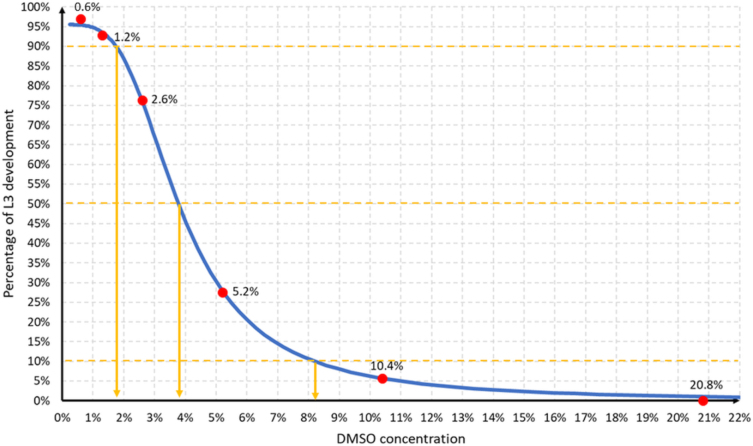
The five-parameter logistic curve (blue line) fitted to the observed percentage development of larvae of the third stage (L3) of *Haemonchus contortus, Trichostrongylus* spp. and *Oesophagostomum* spp. at increasing dimethyl sulfoxide (DMSO) concentrations. Red dots – exact values of tested concentrations indicated. Orange broken lines and arrows – DMSO concentrations inhibiting the development of 10%, 50% and 90% of L3

The PD significantly decreased with increasing DMSO concentration (P < 0.001). In the control wells, the PD ranged from 87.5% to 100% (95.5% ± 3.1%) and remained equally high at the 0.6% and 1.3% DMSO concentrations. At the 2.6% DMSO concentration, it dropped by 16.5% ± 9.4%, and this DMSO concentration was the first at which the PD proved to be significantly reduced compared to that of the control wells (P < 0.001) (Table 1). Subsequently, the decrease in PD was very rapid and between the DMSO concentrations of 2.6% and 5.2% it dropped by another 48.8% ± 13.5% (Fig. 1). The relationship between the DMSO concentration and the PD was modelled by the 5PL curve with the following parameters: lower asymptote (a) = 0%, upper asymptote (d) = 95.52%, Hill’s slope (b) = 3.72, inflection (c) = 3.3% and asymmetry (g) = 0.66. The IC_50_ was 3.79%, the IC_10_ was 1.75% and the IC_90_ was 8.20% (Fig. 2).

**Table 1.** The observed percentage of eggs, larvae of the first and second stage (L1 and L2) and larvae of the third stage (L3; percentage development – PD) at increasing dimethyl sulfoxide (DMSO) concentrations

DMSO concentration		Percentage of developmental stages observed after the 7-day incubation (%) ^a^					
	n	L3 (PD)		Unhatched eggs		L1 and L2	
			P-value ^b^		P-value ^b^		P-value ^b^
0.0% (control)	30	95.5 ± 3.1 (87.5–100)	-^c^	0.05 ± 0.28 (0–1.54)	-^c^	4.43 ± 3.08 (0–12.5)	-^c^
0.6%	30	97.0 ± 2.6 (89.2–100)	0.856	0.99 ± 1.60 (0–8.11)	0.022*	1.99 ± 2.26 (0–7.9)	0.450
1.3%	30	92.8 ± 4.4 (81.0–100)	0.335	0.85 ± 1.27 (0–4.11)	0.068	6.35 ± 4.40 (0–19.0)	0.683
2.6%	30	76.3 ± 8.8 (50.0–91.8)	<0.001*	0.49 ± 0.90 (0–3.13)	0.566	23.2 ± 8.8 (8.2–50.0)	<0.001*
5.2%	30	27.6 ± 10.4 (10.8–50.0)	<0.001*	0.74 ± 1.43 (0–5.41)	0.157	71.7 ± 10.5 (50.0–89.2)	<0.001*
10.4%	30	5.7 ± 6.6 (0–33.7)	<0.001*	1.21 ± 1.53 (0–5.97)	0.003*	93.1 ± 6.6 (66.3–100)	<0.001*
20.8%	30	0.04 ± 0.22 (0–1.2)	<0.001*	0.84 ± 1.27 (0–4.62)	0.073	99.1 ± 1.3 (95.4–100)	<0.001*

a – presented as the arithmetic mean ± standard deviation (range);

b – Dunnett’s post-hoc test versus the control wells (0% concentration);

c – reference category;

* – significant at α = 0.05

With increasing DMSO concentration and decreasing PD, the percentage of L1 and L2 observed in the wells significantly increased (P < 0.001) (Fig. 1). It followed an analogical but opposite pattern to that of the PD, with the first significant increase of the L1 and L2 percentage observed at the 2.6% concentration (Table 1). The L1 and L2 percentage was virtually complementary to that of the PD at each DMSO concentration, with a minimal percentage left for the unhatched eggs. The percentage of unhatched eggs rarely exceeded 1% and also proved to change significantly between DMSO concentrations (P = 0.017) and elevate significantly at the concentrations of 0.6% and 10.4% (Table 1). The observed changes in the percentages of unhatched eggs did not follow any pattern (Fig. 1).

In the culture of pooled faecal samples from the two goats, the L3 of *H. contortus* accounted for 62%, *Trichostrongylus colubriformis* for 35%, and *Oesophagostomum* spp. for 3% of 100 developed larvae counted, while the L3 of *Teladorsagia circumcincta* were not detected. Very similar results were obtained in the control wells in the LDT: *H. contortus* L3 accounted for 52%, *Trichostrongylus* spp. L3 for 46%, and *Oesophagostomum* spp. L3 for 2%. Similar values were also obtained at the 0.6% DMSO concentration.Table 1. The observed percentage of eggs, larvae of the first and second stage (L1 and L2) and larvae of the third stage (L3; percentage development - PD) at increasing dimethyl sulfoxide (DMSO) concentrations.

**Fig. 3.**
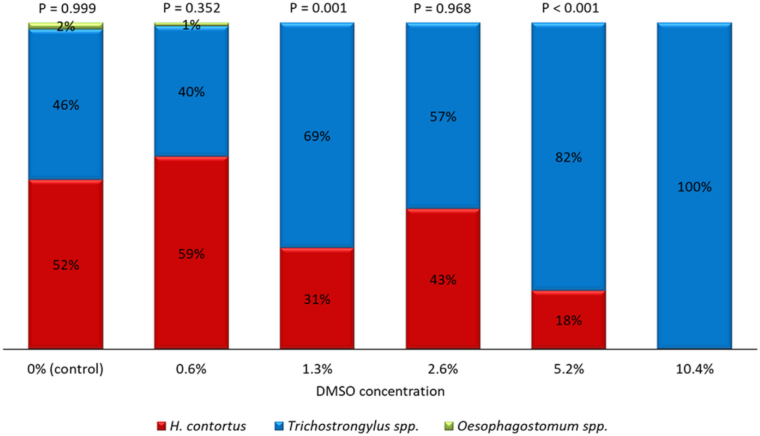
The proportion of developed larvae of the third stage (L3) of *Haemonchus contortus, Trichostrongylus* spp. and *Oesophagostomum* spp. (out of 100 counted L3) at increasing dimethyl sulfoxide (DMSO) concentrations. P-values of McNemar’s test

The first change was observed at the 1.3% DMSO concentration – no *Oesophagostomum* spp. L3 were found and *Trichostrongylus* spp. L3 significantly outnumbered *H. contortus* L3 (P = 0.001). At the 2.6% DMSO concentration, the difference between the proportions of *H. contortus* and *Trichostrongylus* spp. was insignificant (P = 0.968), but then the share of *H. contortus* became significantly lower again at the 5.6% DMSO concentration (P < 0.001), and at the 10.4% DMSO concentration no *H. contortus* L3 were found at all (Fig. 3).

## Discussion

Our study clearly shows that DMSO inhibits the *in vitro* development of L3 (the transition from L1 and L2 to L3) in *H. contortus, Trichostrongylus* spp. and *Oesophagostomum* spp. This effect became statistically significant at the DMSO concentration of 2.6%; however, PD fell below 90% at a concentration as low as 1.75%. The L3 of *H. contortus*, and probably also of *Oesophagostomum* spp., appeared to be more susceptible to DMSO than those of *Trichostrongylus*. At the same time, DMSO did not seem to exert an *in vitro* ovicidal effect (the inhibition of transition from eggs to L1) in these GIN.

The concept of this study resulted from attempts which we had made to improve the LDT by using various concentrations of anthelmintics. This required some manipulations of the concentration of DMSO used as a drug solvent (vehicle). The inhibitory effect of DMSO on larval development may substantially confound the results of an LDT, especially when DMSO is only used as a solvent in wells containing tested anthelmintics and not in the control wells. This observation highlights the importance of careful selection of a suitable concentration of DMSO solvent in experiments investigating anthelmintic drug efficacy in GIN of the Trichostrongylidae family.

The chemical nature of DMSO allows it to interact with various chemical compounds and promote solubility of hydrophobic molecules. It is known, however, that DMSO used as a solvent can impair the integrity of biological systems, even though these effects usually become apparent at much higher concentrations than those typically used in laboratory experiments (5). We observed the first significant PD reduction at the DMSO concentration of 2.6%; however, the decrease in PD which we observed between 1.3% and 2.6% was so considerable that we recommend the concentration of 2.6% not be considered as the lowest DMSO concentration detrimental to L3. In our opinion, the IC_10_ of 1.75% is a reasonable threshold value. This finding is of value moving forward, as although the concentrations of DMSO used for LDT do not normally exceed 1.75%, this study nonetheless represents the first example of testing the effects of higher DMSO concentrations on parasitic nematodes. This is of importance, as although the effects of higher DMSO concentrations have been explored in *C. elegans* (5), there are significant differences between the physiology of free living and of parasitic nematodes. This can have important implications when considering AR (48), and by extension tests which are used to determine susceptibility to anthelmintic drugs. Given that the standard DMSO concentration used in the LDT is 1.3%, our study indicates that still using additional control wells containing water instead of DMSO for water-soluble drugs used in LDT (such as LEV) is not necessary. This observation may allow for a reduction in the time and effort associated with performing LDT and reading the results.

The mechanisms *via* which various chemical compounds (including DMSO) affect nematodes have mostly been studied in *C. elegans* (5, 7, 31). The major mechanism appears to involve the impairment of the ability to feed, which relies on the activity of the pharyngeal system (pharyngeal pumping) (5, 24, 47). In our experiment, we did not observe any ovicidal effect of DMSO on the trichostrongylid eggs. At increasing DMSO concentrations, the vast majority of eggs still hatched and released larvae. With increasing DMSO concentration, the L1-and-L2-to-L3 ratio increased substantially, while the egg percentage remained very low. As only L1 and L2 actively feed, it may indicate a leading role of the effect of DMSO on pharyngeal activity. However, we cannot exclude a morphological change-inducing effect of DMSO altering internal body composition, which can arise during prolonged exposure and which we did not measure in our experiment.

This observation suggests that DMSO is unlikely to affect results of *in vitro* tests (*e.g*. EHT) which measure the ovicidal effect of BZ by calculating the ratio of unhatched eggs to L1 and L2 and does not require the development of larvae to the L3 stage. Calahorro *et al*. (5) proved that 1% DMSO concentration inhibited pharyngeal pumping in the adult stage (L4) of *C. elegans* after 3 h of exposure; however, pumping reverted to pre-exposure levels after the *C. elegans* L4 were continually exposed to the DMSO at the same concentration. Although the physiology and life cycle of *C. elegans* differ from parasitic nematodes, this phenomenon may account for the ability of GIN to survive and develop at certain DMSO concentrations, especially during anthelmintic activity tests in which incubation and observation are carried out for several days *e.g*. up to 7 days of incubation in the LDT. Many anthelmintic drugs (including ML and LEV) show an *in vitro* effect similar to the DMSO effect, *i.e*. they inhibit transition of L1 and L2 into L3 by disrupting the pharyngeal pumping mechanism (47). Therefore, it is crucial to carry out experiments in a way which ensures that the observed PD reduction reflects the true influence of the tested drugs and not that of the vehicles and solvents such as DMSO.

The main limitation of the study was the examination of the effect of DMSO on larval development in only three GIN species or genera. It would be appropriate also to determine the effect of DMSO on other species of GIN, especially on *T. circumcincta*, which is the most widespread GIN in mild climates and is also one of the three most pathogenic species in the Trichostrongylidae family occurring on small ruminant farms (51). Therefore, further studies should be carried out with a larger number of animals and a more diverse GIN species composition, for acquisition of overall data on the effect of DMSO on larval development in the LDT.

## Conclusion

Our study showed that DMSO at the concentration of 2.6% significantly inhibited the development of the L3 of *H. contortus, Trichostrongylus* spp. and *Oesophagostomum* spp., and that a practically important inhibitory effect (IC10) may be observed even at a concentration as low as 1.75%. DMSO at concentrations below 1.75% did not appear to inhibit larval development. On the other hand, DMSO did not seem to exert an *in vitro* ovicidal effect on these GIN, regardless of the concentration in which it is used.
